# Perspectives in newborn screening for SCID in Japan. Case report: newborn screening identified X-linked severe combined immunodeficiency with a novel *IL2RG* variant

**DOI:** 10.3389/fimmu.2024.1478411

**Published:** 2024-11-20

**Authors:** Shiro Beppu, Takuro Nishikawa, Dan Tomomasa, Atsushi Hijikata, Hiroshi Kasabata, Hideyuki Terazono, Kazuro Ikawa, Tatsuro Nakamura, Shogo Horikawa, Jun Nagahama, Aki Nakamura, Takanari Abematsu, Shunsuke Nakagawa, Kaoru Oketani, Hirokazu Kanegane, Yasuhiro Okamoto

**Affiliations:** ^1^ Department of Pediatrics, Graduate School of Medical and Dental Sciences, Kagoshima University, Kagoshima, Japan; ^2^ Department of Pediatrics and Developmental Biology, Graduate School of Medical and Dental Sciences, Tokyo Medical and Dental University (TMDU), Tokyo, Japan; ^3^ School of Life Sciences, Tokyo University of Pharmacy and Life Sciences, Tokyo, Japan; ^4^ Department of Clinical Laboratory Medicine, Kagoshima University Hospital, Kagoshima, Japan; ^5^ Department of Clinical Pharmacy and Pharmacology, Graduate School of Medical and Dental Sciences, Kagoshima University, Kagoshima, Japan; ^6^ Department of Clinical Pharmacotherapy, Hiroshima University, Hiroshima, Japan; ^7^ Kagoshima Prefectural Comprehensive Health Centre, Kagoshima, Japan; ^8^ Department of Child Health and Development, Graduate School of Medical and Dental Sciences, Tokyo Medical and Dental University (TMDU), Tokyo, Japan

**Keywords:** hematopoietic stem cell transplantation, *IL2RG* gene, newborn screening, structural analysis, X-linked severe combined immunodeficiency

## Abstract

**Background:**

Newborn screening (NBS) for severe combined immunodeficiency (SCID) has improved the prognosis of SCID. In Japan, NBS testing (measurement of the T-cell receptor excision circles (TREC) and kappa-deleting recombination excision circles (KREC)) was launched in 2017 and has expanded nationwide in recent years. In this study, we report a Japanese patient with X-linked SCID with a novel *IL2RG* variant identified through NBS. The patient underwent cord blood transplantation (CBT).

**Case:**

The patient had no siblings or family history of inborn errors of immunity. He was born at 38 weeks of gestation and weighed 3,072 g. His NBS results revealed TREC 0 copies/10^5^ cells (normal value: >565 copies/10^5^ cells), which was considered suggestive of SCID. The patient was referred to our hospital. Although his lymphocyte count was 1,402/μL, naïve T cells and CD56^+^ natural killer (NK) cells were decreased to 0% and 0.05% of the total lymphocytes, respectively. Flow cytometric measurement testing revealed a decrease in γc protein expression in the B lymphocytes and NK lymphocytes. We identified a hemizygous novel missense variant (c.256A>C, p.Thr86Pro) of *IL2RG*. Both *in silico* and structural analyses revealed that this variant is likely pathogenic. At 3 months of age, he underwent CBT from a human leukocyte antigen-full-matched unrelated donor. The conditioning regimen included fludarabine (180 mg/m^2^) and targeted busulfan (35 mg×h/L). The patient achieved high-level donor chimerism and immune reconstitution, including B-cell function, at 13 months.

**Conclusion:**

Using NBS, the patient was diagnosed as having X-linked SCID with a novel missense variant of *IL2RG*. Early diagnosis using NBS tests enables safe hematopoietic stem cell transplantation without complications such as infection. We also found that even SCID with novel variants can be accurately diagnosed using the NBS program. In Japan, the test uptake rate is approximately 80% due to the high number of self-funded screening tests, and it is hoped that the uptake rate will increase in the future.

## 1 Introduction

Severe combined immunodeficiency (SCID) is a rare and fatal inborn error of immunity (IEI) that is mainly caused by T- and B-cell differentiation disorders and develops in early infancy with severe infections (1, 2). Pathogenic variants have been identified in more than 20 genes that cause classical or leaky SCID phenotypes (3, 4). The most common genetic cause of SCID is variants in *IL2RG* (X-linked SCID), which encodes for the common gamma chain (γc) of the interleukin-2 (IL-2) receptor. The common γc is also shared by leukocyte receptors for other cytokines (IL-4, IL-7, IL-9, IL-15, and IL-21) that are relevant in T cell, natural killer (NK) cell, and memory B cell development; therefore, most patients have a T−B+NK−SCID phenotype (3). Hematopoietic stem cell transplantation (HSCT) is the standard curative treatment for SCID. Early HSCT before severe infection or live vaccination is an important factor for improving the prognosis of SCID (5, 6). The T-cell receptor excision circle (TREC), circular double-stranded DNA produced by rearrangement of the T-cell receptor α gene, is a molecular marker developed in 2005 that sensitively reflects normal T lymphopoiesis. In the United States, newborn screening (NBS) for SCID using the TREC quantitative polymerase chain reaction (PCR) assay proved to be successful in pilot studies starting in 2008, and by 2019, all states had contributed to an improved prognosis (7). The kappa-deleting recombination excision circle (KREC), a circular double-stranded DNA produced during B-cell receptor rearrangement, is a marker of normal B cell lymphopoiesis and was developed in 2007. In Japan, optional NBS (TREC and KREC assays) was launched in Aichi Prefecture in 2017 and is spreading to all prefectures (8). Herein, we report a case of a Japanese patient with X-linked SCID with a novel *IL2RG* variant identified through NBS.

## 2 Case description

Informed consent was obtained from the parents of the patient. The study was conducted in accordance with the principles of the Helsinki Declaration and the protocol was approved by the Ethics Committee on Clinical Research, Sakuragaoka Campus, Kagoshima University.

**Figure 1:**
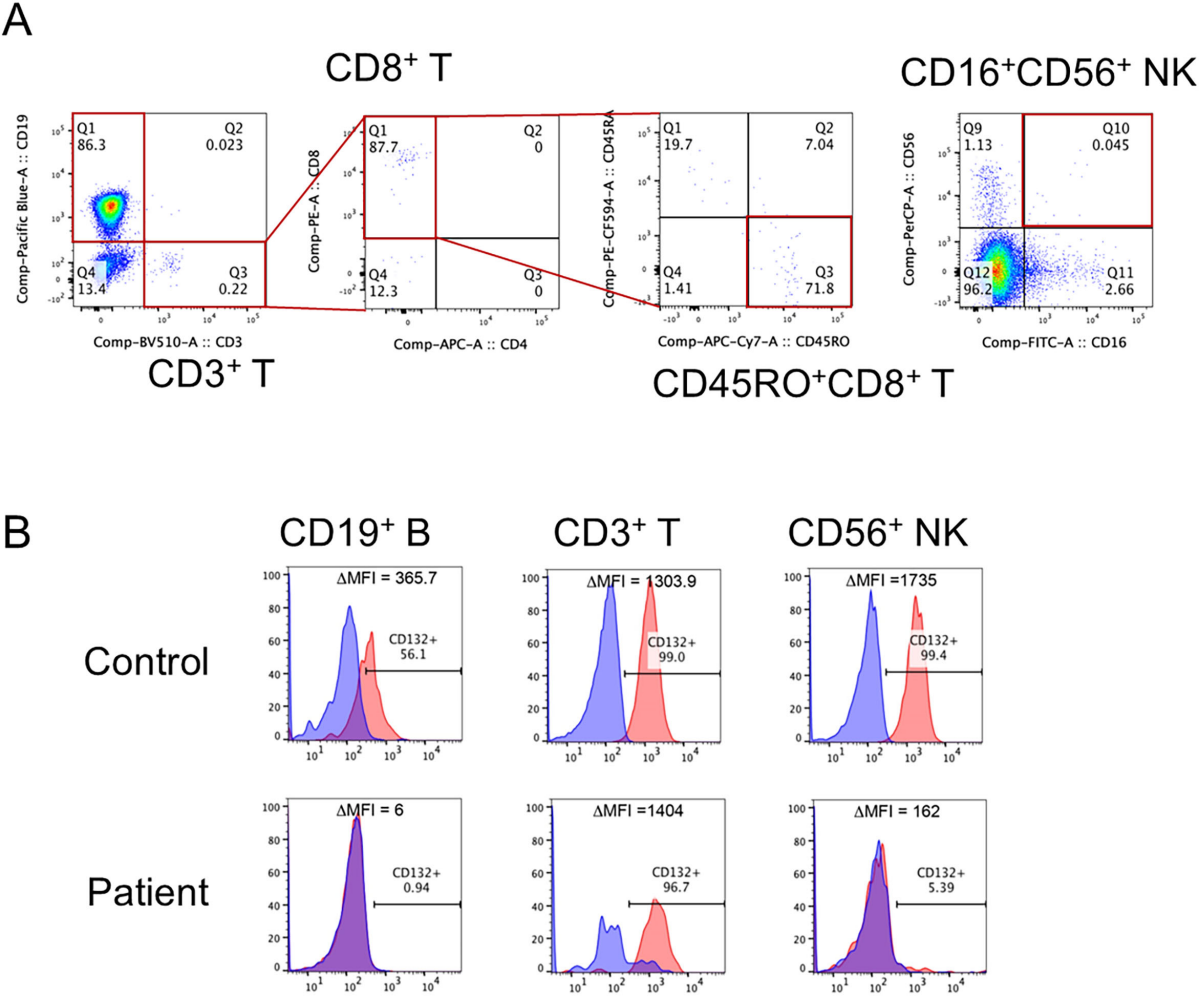
Flow cytometric analysis of lymphocyte subpopulations and expression of γc in our patient. **(A)** CD3^+^ T cells and CD56^+^ natural killer (NK) cells were decreased to 0.2% and 0.05% of the total lymphocytes, respectively. The CD3^+^ T cells were mostly CD45RO^+^CD8^+^ T cells (memory CD8^+^ T cells). **(B)** The expression of γc was lacking in CD19^+^ B cells and CD56^+^ NK cells.

The patient had no siblings, family history of IEI, or early childhood death. He was born at 38 weeks of gestation via caesarean section and weighed 3,072 g without prenatal or delivery complications. NBS for SCID showed TREC 0 copies/10^5^ cells (normal value: >565 copies/10^5^ cells) and KREC 4,473 copies/10^5^ cells (normal value: >456 copies/10^5^ cells), which was considered suggestive of SCID. The patient was subsequently referred to our hospital for immunological evaluation. Vital signs and physical findings were unremarkable. His white blood cell count was 5,840/μL with 56% granulocytes and 24% lymphocytes (1,402/μL). The serum levels of IgG, IgA, and IgM were 846, <4, and 11 mg/dL, respectively (normal ranges: 236–1104, 10–70, and 15–110 mg/dL, respectively). In the analysis of lymphocyte subpopulations, CD3^+^ T cells and CD56^+^ natural killer (NK) cells were decreased to 0.2% (normal, 49–76%) (2.8/μL) and 0.05% (normal, 6.5–11.5%) (0.7/μL) of the total lymphocytes, respectively (**Figure 1A**), and CD19^+^ B cells were increased to 97% (normal, 9–16%) (1,359.6/μL). The few CD3^+^ cells were mostly memory CD8^+^(CD45RO^+^) cells and were considered maternal T cells (**Figure 1A**) because γc protein expression (CD132) was observed in the T cells (**Figure 1B**). In the lymphocyte stimulation test, proliferation in response to both phytohemagglutinin (PHA) stimulation and concanavalin A was low (1,171 cpm; normal value: 20,500–56,800 cpm and 1,174 cpm; normal value: 20,300–65,700 cpm, respectively). A chest radiograph revealed a thymic shadow defect. Additionally, flow cytometric measurement testing revealed a decrease in γc protein expression in B lymphocytes and NK lymphocytes (**Figure 1B**). A phenotype of T−B+NK−SCID was determined based on Primary Immune Deficiency Treatment Consortium (PIDTC) criteria and the patient was referred for HSCT evaluation (9).

### 2.1 Genetic analysis

**Figure 2:**
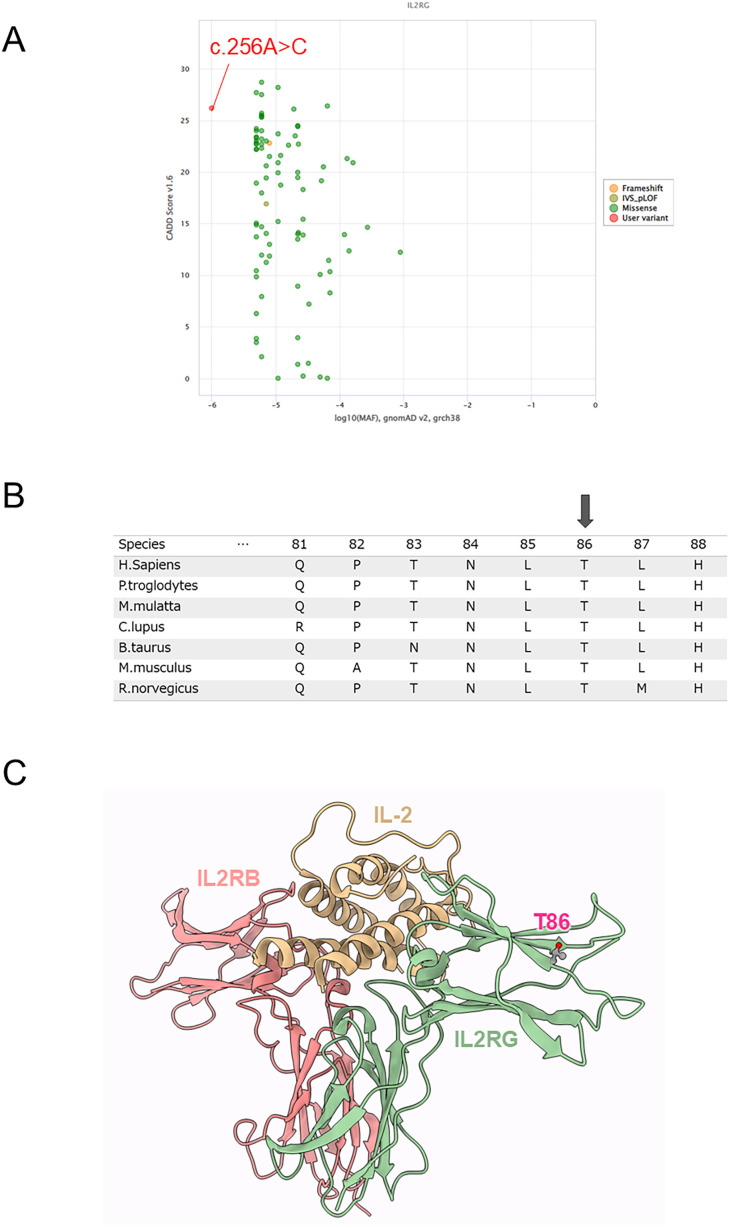
Genetic and protein structure analysis. **(A)** In the *in silico* analysis of the *IL2RG* variant, the minor allele frequency (MAF) and Combined Annotation-Dependent Depletion (CADD) scores of *IL2RG* were -6 and 26.2 in the c.256A>C variant, respectively. **(B)** Amino acid sequence alignment of *IL2RG* among species. Arrow indicates a missense variant (p.Thr86Pro). **(C)** Structural analysis of IL2RG protein. Structure of IL-2 and IL-2 receptor complex (PDB code: 5m5e). Thr86 was located in the β-sheet of the Ig-like domain that binds IL-2; substitutions for Pro generally tend to break the β-sheet and destabilize the structure.

Targeted panel sequencing of SCID (*IL2RG, JAK3, IL7R, RAG1, RAG2, DCLRE1C, ADA, PNP, ZAP70, LIG4, NHEJ1*, and *TBX1)* was performed at the Kazusa DNA Research Institute using genomic DNA from peripheral blood mononuclear cells. We identified a novel hemizygous missense variant (c.256A>C, p.Thr86Pro) of *IL2RG*. In the *in silico* analysis of the *IL2RG* variant, the minor allele frequency and Combined Annotation-Dependent Depletion scores of *IL2RG* were -6 and 26.2 in the c.256A>C variant, respectively (**Figure 2A**). In the amino acid sequence alignment of *IL2RG* among species, the amino acids in the novel variant were conserved in all species (**Figure 2B**).

### 2.2 Protein structure analysis

The atomic coordinates of the IL-2 in complex with the IL-2 receptors in humans were obtained from the Protein Data Bank (PDB) (10). The PDB contained 3D structures of IL-2 in complex with the IL-2 receptor, namely, the crystal structure of an IL-2 variant in complex with the IL-2 receptor (PDB code: 5m5e). Thr86 was located in the β-sheet of the Ig-like domain that binds IL-2; substitutions for Pro generally tend to break the β-sheet and destabilize the structure (**Figure 2C**). Changes in the thermal stability of the IL-2 receptor domain in response to the missense variant were evaluated using FoldX version 4 (11). The energy change of the p.Thr86Pro variant was +1.6 kcal/mol, suggesting the variant would destabilize the structure.

### 2.3 Patient’s treatment course and follow-up

The patient was managed in a sterile room and started on a prophylactic oral sulfamethoxazole-trimethoprim combination for *Pneumocystis* pneumonia from 1 month of age. His IgG level decreased to 447 mg/dL and immunoglobulin replacement therapy was initiated at 2 months of age. There were no signs of infection, and the patient’s weight increased steadily. At 3 months of age, he underwent cord blood transplantation (CBT; nucleated cell count 24.5×10^7^/kg, CD34^+^ cell count 8.9×10^5^/kg) from a human leukocyte antigen-fully matched unrelated female donor. The conditioning regimen included fludarabine (30 mg/m^2^; days -7 to -2) and targeted busulfan (4 mg/kg; days -4 and -2) (total area under the blood-concentration-time curve 35 mg×h/L). Tacrolimus and short-term methotrexate were administered as prophylaxis for acute graft-versus-host disease (GVHD). Neutrophil engraftment was achieved 14 days after transplantation. High-level mixed chimerism was documented using XY chromosome fluorescein *in situ* hybridization at month 1 (81%), 3 (88%), 6 (85%), and 12 (83%). The patient’s CD3^+^, CD4^+^, and CD19^+^ lymphocyte counts and percentage of CD45RA^+^CD4^+^ T lymphocytes increased steadily, and it reached a plateau after CBT and were 927/µL, 278/µL, 2101/µL, and 16%, respectively, at 2 months after transplantation. At 1 year after the transplantation, the patient’s CD3^+^, CD4^+^, and CD19^+^ lymphocyte counts, percentage of CD45RA^+^CD4^+^ T lymphocytes, and IgG/A/M immunoglobulin level were 2,123/µL, 1,704/µL, 997/µL, 36.8%, and 726/121/238 mg/dL, respectively. Furthermore, immunoglobulin replacement therapy was not required from 2 months after transplantation to the present (13 months after transplantation).

From 73 days after CBT, the patient had a cough, and his C-reactive protein level was mildly elevated. Computed tomography revealed granular and infiltrative shadows mainly in the right lung and thickening of the bronchial wall. A comprehensive PCR test for respiratory microorganisms using a nasopharyngeal swab yielded negative results. He was diagnosed with noninfectious pulmonary complications and administered methylprednisolone from 96 days after CBT. The lung lesions and coughing rapidly improved. The steroid dose was tapered off, and the patient was discharged 126 days after CBT. Currently, 13 months after CBT, the patient is progressing without GVHD or complications, and the tacrolimus dose has been tapered off.

### 2.4 tSNE analysis

**Figure 3:**
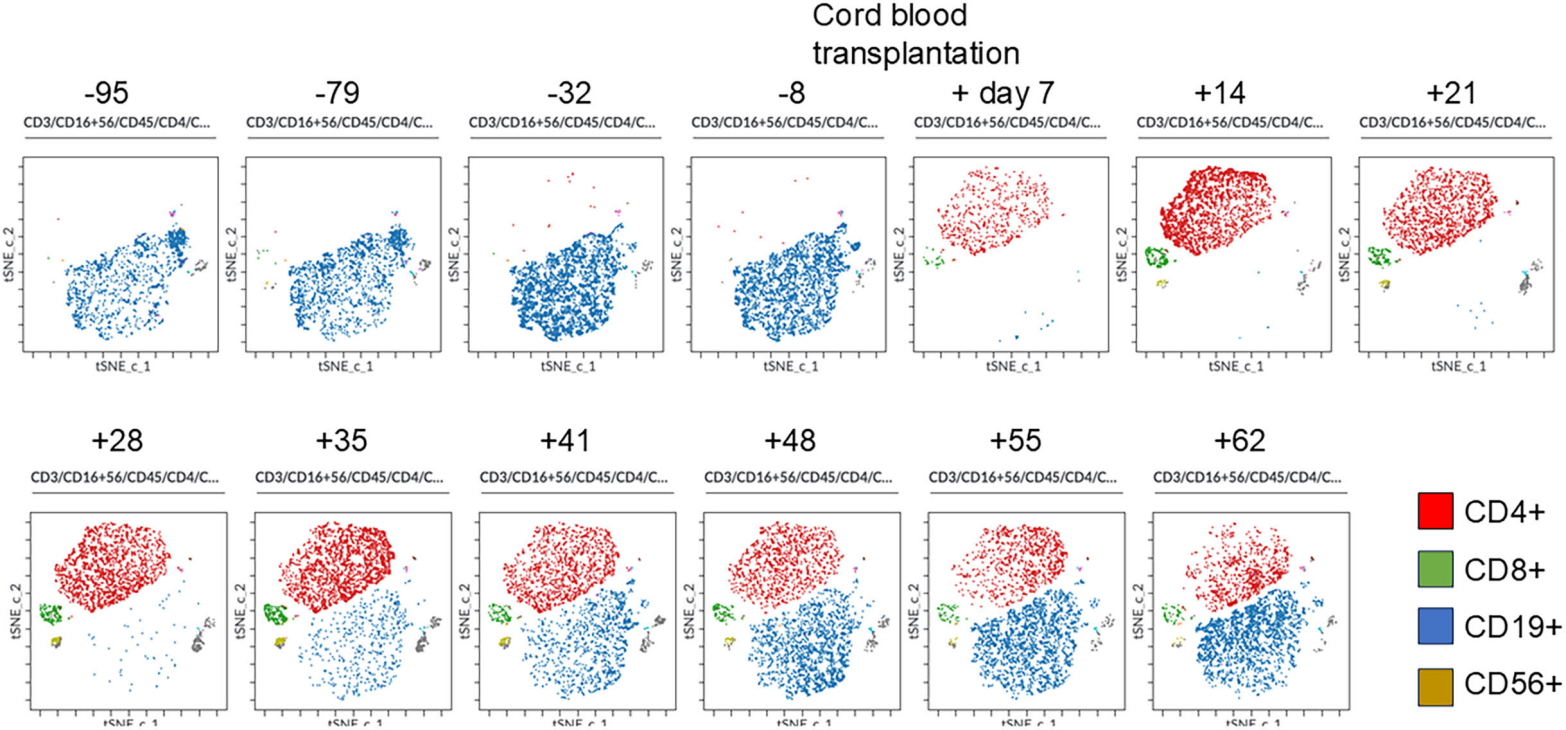
Changes in lymphocyte and natural killer cell populations by tSNE-CUDA analysis before and after cord blood transplantation. Peripheral blood mononuclear cells were chronologically analyzed using flow cytometry with six colors (CD45, CD3, CD16/CD56, CD4, CD19, and CD8). Standard flow cytometry files were subjected to graphics processing-unit-accelerated t-distributed stochastic neighbor embedding (tSNE-CUDA) analysis using Cytobank. The tSNE-CUDA analysis allowed us to dynamically capture how each lymphocyte subset was engrafted and how it increased after CBT in our patient with X-linked SCID. CD4^+^ cells were first established, followed by CD8^+^, CD56^+^, and CD19^+^ cells, which increased in number.

Peripheral blood mononuclear cells were chronologically analyzed using flow cytometry with six colors (CD45, CD3, CD16/CD56, CD4, CD19, and CD8). Standard flow cytometry files were subjected to graphics processing-unit-accelerated t-distributed stochastic neighbor embedding (tSNE-CUDA) analysis using Cytobank™ (Beckman Coulter Life Sciences, Indianapolis, IN). The tSNE-CUDA analysis allowed us to dynamically capture how each lymphocyte subset was engrafted and how it increased after CBT in our patient with X-linked SCID. CD4^+^ cells were established first, followed by CD8^+^, CD56^+^, and CD19^+^ cells, which increased in number (**Figure 3**).

## 3 Discussion

The PIDTC in the United States transplant data of 36 years and 902 patients with SCID reported a marked improvement in overall survival only in the period 2010-2018, the era of initiation and expansion of NBS (7). Additionally, the 5-year overall survival rate of patients diagnosed with SCID through NBS after 2010 is 92.5%, and they have a better prognosis than do patients diagnosed based on family history (79.9%) or clinical symptoms (85.4%) (7). Thus, the usefulness of NBS for SCID remains unequivocal.

The first NBS program in Japan, in Aichi Prefecture, started in 2017, and 137,484 newborns were screened by December 2021, with 145 (0.11%) having abnormal TREC/KREC values; two SCID cases (X-linked SCID and reticular dysgenesis) were ultimately diagnosed (8). In Kagoshima Prefecture, SCID NBS tests were started in July 2022, and 14,099 newborns were screened by March 2024, with 14 (0.1%) showing abnormal TREC/KREC values; one SCID case (present case: X-linked SCID) was finally diagnosed. In Japan, SCID NBS tests have expanded rapidly in recent years, and as of March 2024, 40 among 47 prefectures were conducting these tests (12). However, at present in Japan, most facilities require patients to pay for NBS tests for SCID. To improve the uptake rate of NBS, it is desirable for screening tests to be covered by public funds. Recently, demonstration projects have begun by national governments and, in some prefectures, the cost of the tests is being covered by public funds.

The present case was a novel missense variant of *IL2RG*; however, both *in silico* and structural analyses suggested that it was a pathological variant. Therefore, this novel variant was diagnosed as a pathological variant. After implementation of the NBS program, SCIDs of genotypes other than X-linked SCID have been diagnosed more frequently (13). Physicians should recognize this in patients diagnosed with SCID by using NBS programs. We also found that even SCID with novel variants, as in the present case, can be accurately diagnosed using the NBS program.

After SCID diagnosis, the patient was admitted to the hospital and managed in a sterile room for infection control until transplantation. Depending on the medical situation in each country, proactive approaches such as lifestyle guidance, infection surveillance, and extensive prophylaxis should be used to prevent infection in infants until the transplantation treatment is performed (14). In a patient with SCID identified through NBS, the child appears completely normal; therefore, the family may be concerned or reluctant to have the child undergo HSCT. In our case, the medical staff understood the family’s struggles and provided easy-to-understand explanations about the disease, the risks that could occur if left untreated, and HSCT. Additionally, the medical staff answered minor questions on a daily basis until HSCT, and built a relationship of trust with the patient’s family. As we planned to perform HSCT using conditioning, we waited until 3 months of age to perform CBT for safety reasons. There is still no consensus regarding how long to wait for HSCT with conditioning after a SCID diagnosis through NBS. In this case, reduced intensity conditioning, fludarabine, and targeted busulfan (35 mg×h/L) were chosen because they are safe and effective strategies for obtaining high-level donor chimerism, immune reconstitution including B-cell function, and long-term survival in patients with SCID (15). We performed lineage-specific chimerism by droplet digital PCR for sex-determining region Y gene (Y-linked genes) at 2 months after CBT (16). The results showed donor-type chimerism of 90.8% in whole blood cells, 88.2% in the granulocyte fraction, 92.9% in mononuclear cells, 99.1% in T cells, and 93.2% in non-T cells (**Supplementary Figure 1**). CBT was performed safely with no serious adverse events, including infections. The patient developed a noninfectious pulmonary complication, which quickly resolved mildly and was otherwise unremarkable.

Using tSNE analysis, we visually and dynamically captured the respective lymphocyte subset population viability after HSCT in patients with SCID for the first time. In the present case, B-cell neoplasia was observed earlier, probably due to CBT (17). Accumulation of serial data by tSNE analysis of lymphocyte subsets of patients undergoing HSCT for SCID may predict the risk of poor engraftment and development of GVHD. Even if they appear to be in the same subset population, differences can be found using tSNE analysis.

In conclusion, we diagnosed X-linked SCID with a novel missense *IL2RG* variant using the TREC/KREC NBS test. Early diagnosis using NBS tests allowed for safe HSCT without complications, including infection. In Japan, the uptake rate of the test is 80-90% because the test is often self-funded. It is hoped that the uptake rate will be increased in the future through public funding.

## Data Availability

The raw data supporting the conclusions of this article will be made available by the authors, without undue reservation.
