# Supplementary material for: Perspectives in newborn screening for SCID in Japan. Case report: newborn screening identified X-linked severe combined immunodeficiency with a novel IL2RG variant
Source: Front Immunol. 2024 Nov 20;15:1478411. doi: 10.3389/fimmu.2024.1478411 (PMC11614797; doi:10.3389/fimmu.2024.1478411)
Supplement: Supplementary file 1 [file DataSheet1.docx]

Supplementary Material


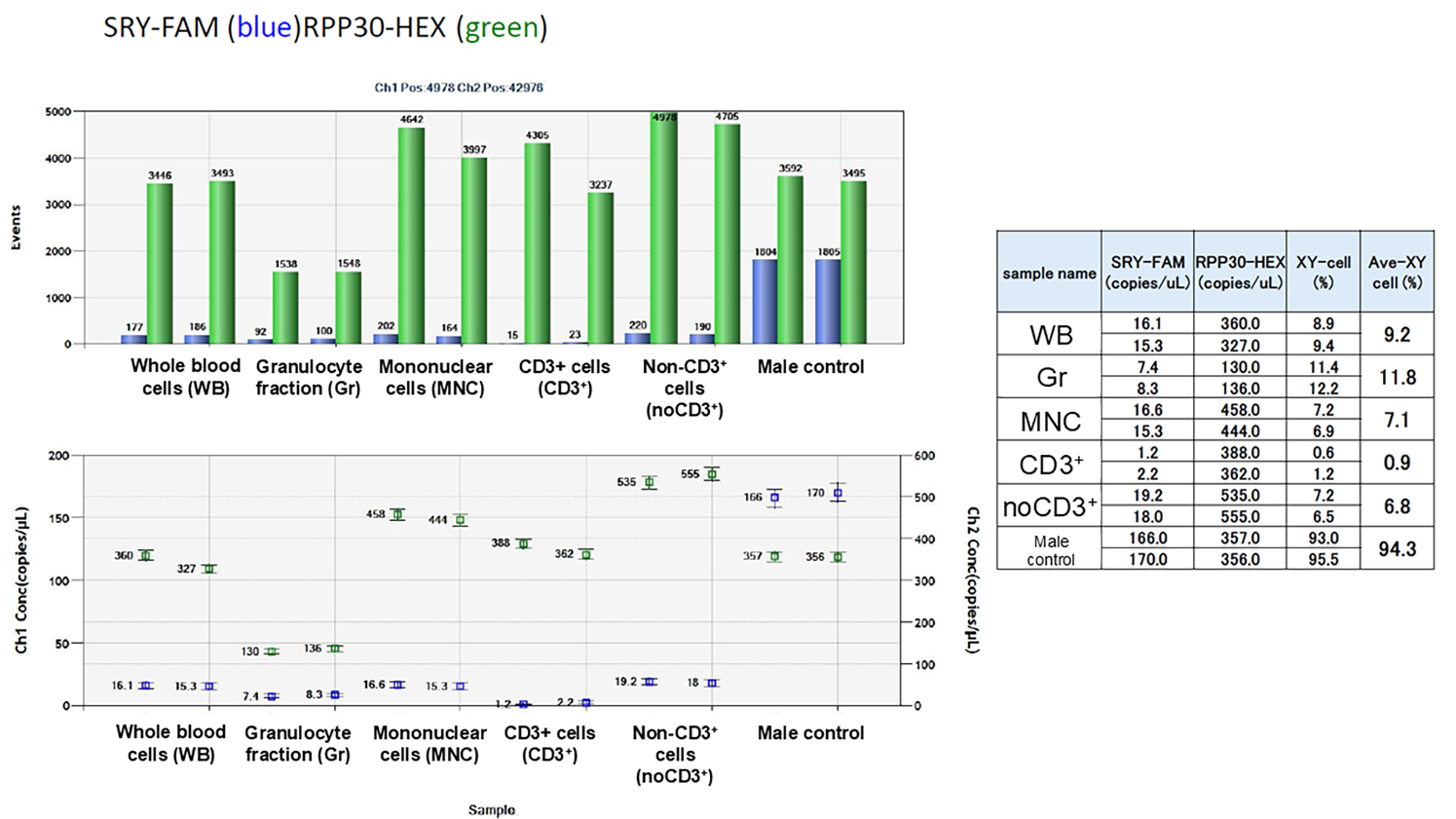


**Supplementary Figure 1.** **Lineage-specific chimerism by droplet digital PCR for sex-determining region Y gene (Y-linked genes) at 2 months after cord blood transplantation**

The results showed donor-type chimerism of 90.8% in whole blood cells, 88.2% in the granulocyte fraction, 92.9% in mononuclear cells, 99.1% in T cells, and 93.2% in non-T cells.
